# Resveratrol’s Neuroprotective Effects on the Retina in a Pentylenetetrazol-Induced Epilepsy Model in Rats: Insights into SIRT1 Signaling, Apoptosis, and Gliosis

**DOI:** 10.3390/antiox15091168

**Published:** 2026-09-15

**Authors:** Omer Unal, Nilufer Akgun-Unal, Elif Gulbahce-Mutlu, Seda Simsek, Mustafa Ayyildiz

**Affiliations:** 1 Department of Physiology, Faculty of Medicine, Samsun University, 55080 Samsun, Türkiye; 2 Department of Biophysics, Faculty of Medicine, Ondokuz Mayis University, 55139 Samsun, Türkiye; 3 Department of Medical Biology, Faculty of Medicine, KTO Karatay University, 42020 Konya, Türkiye; 4 Department of Histology and Embryology, Faculty of Medicine, Selcuk University, 42130 Konya, Türkiye; 5 Department of Physiology, Faculty of Medicine, Ondokuz Mayis University, 55139 Samsun, Türkiye

**Keywords:** epilepsy, retina, resveratrol, SIRT1, GFAP, apoptosis, PTZ

## Abstract

Background/Objectives: A chronic neurological disorder known as epilepsy results in neurodegeneration, oxidative stress, and damage to the retina and the central nervous system’s structure. The main objective of this research was to examine the neuroprotective benefits of Resveratrol (RES) on the retina in a model using Pentylenetetrazol (PTZ) in rats to induce kindling and to investigate the underlying molecular and biochemical mechanisms (SIRT1, GFAP, VEGF, apoptotic pathways, and oxidative stress markers [malondialdehyde (MDA) and reduced glutathione (GSH)] of this protective effect. Methods: A total of thirty-two male Wistar albino rats were randomly assigned to four distinct groups: Sham, RES (5 mg/kg/day), PTZ (35 mg/kg), and PTZ + RES (*n* = 8 per group). Electrocorticography (ECoG) recordings were used to assess seizure severity. Retinal expression of GFAP, VEGF, SIRT1, Bax, Bcl-2, Caspase-3, and Caspase-9 was evaluated via qPCR and immunofluorescence, while tissue lipid peroxidation (MDA) and reduced glutathione (GSH) levels were quantified spectrophotometrically to directly evaluate retinal redox status. Results: Substantial increases in seizure scores and electrocorticogram spike counts were noted in the PTZ group, whereas RES treatment significantly reduced the total ECoG spike count by 49.6% (from 702.87 ± 145.82 in PTZ to 354.25 ± 31.22 in PTZ + RES, *p* < 0.0001) and lowered the average seizure stage from 4.25 ± 0.27 to 2.43 ± 0.49 (*p* < 0.05), while lengthening the initial myoclonic jerk latency (from 162 ± 29.62 s to 219.37 ± 32.12 s, *p* < 0.01). Molecular and histopathological analyses revealed that RES treatment was associated with enhanced localized retinal cell survival and a significant increase in the nuclear SIRT1 immunofluorescence area fraction in the retina, which more than doubled the nuclear SIRT1 immunofluorescence area fraction in the retina (from 4.2 ± 0.9% in PTZ to 8.4 ± 0.8% in PTZ + RES, *p* < 0.05) despite a physiological transcription-level feedback reduction in SIRT1 mRNA. Furthermore, RES treatment significantly suppressed PTZ-induced retinal apoptosis (Caspase-3 area fraction decreased from 10.6 ± 0.9% to 5.6 ± 0.8%, *p* < 0.001) and attenuated reactive gliosis (GFAP area fraction decreased from 14.1 ± 1.2% to 9.1 ± 1.4%, *p* < 0.01). Biochemically, PTZ kindling induced a profound increase in retinal lipid peroxidation (MDA) and a severe depletion of reduced glutathione (GSH) reserves, both of which were robustly reversed by RES treatment back toward physiological Sham levels (*p* < 0.01). Conclusions: Chronic epilepsy causes glial activation, oxidative damage, and apoptosis in the retina, with RES-associated retinal protection involving the restoration of redox homeostasis and the upregulation of the SIRT1 signaling pathway. These preclinical findings suggest that RES represents a promising protective strategy against epilepsy-associated retinal damage, though further clinical validation is warranted to establish its therapeutic safety and efficacy in human patients.

## 1. Introduction

A chronic neurological condition known as epilepsy is characterized by recurring, unprovoked seizures which trigger widespread metabolic stress, neuroinflammation, and oxidative damage across the central nervous system [1,2]. The retina is anatomically linked to the brain, with similarities in neurotransmitter systems and microvascular structure, yet its susceptibility to seizure activity and adaptive mechanisms have received relatively little research attention. There is increasing evidence that seizure-induced excitotoxicity, oxidative stress, and inflammatory cascades can damage retinal structure and function, and may compromise the survival of photoreceptors, the integrity of synapses in the inner plexiform layer, and retinal ganglion cells [3]. Scientists have found rises in reactive oxygen species, problems with mitochondria, microglial activation, and leakage across the blood-retinal barrier in rat models of temporal lobe epilepsy and general convulsive disorders (such as those induced by pilocarpine, kainate, and pentylenetetrazole seizures). These mechanisms mirror the neuropathology observed in the hippocampus and cortex, providing a strong rationale for retinal neuroprotection. Recent clinical studies show that chronic seizures and prolonged use of antiseizure drugs can markedly impair the endogenous antioxidant defense system, resulting in both systemic and tissue-specific oxidative damage [4].

Epilepsy may lead to progressive neuronal degeneration over the long term. While various antiseizure medications (ASMs) are clinically prescribed to manage seizures, their long-term use is frequently associated with systemic side effects and the generation of free radicals, which can induce oxidative harm in both the central nervous system (CNS) and the retina [4]. The similarities between the retina and the brain in terms of embryological, morphological, and physiological characteristics suggest that the retina can serve as a diagnostic window to the brain due to neuroinflammation and degeneration manifesting in the ocular microenvironment [5,6]. Alterations in retinal nerve fiber layer (RNFL) and ganglion cell complex (GCC) thickness are widely used clinically as non-invasive biomarkers of retinal structural damage and neurodegeneration in epilepsy patients. The precise molecular cascades underlying these structural changes, such as apoptosis in the ganglion cell layer (GCL) and macroglial activation, remain poorly understood. Investigating the transcriptional and cellular changes in these specific retinal layers in preclinical models is therefore crucial to bridge the gap between clinical observations of RNFL/GCC thinning and the underlying neuropathology.

Changes in the structure of the retina have been used as a sign of disease activity, severity, and progression in various neurological conditions, including multiple sclerosis (MS) [7], migraine [8], Alzheimer’s disease (AD) [9], and Parkinson’s disease (PD) [10,11]. Changes in the RNFL and GCC of epilepsy patients have been proposed as a sensitive indicator for monitoring disease outcomes [12].

Resveratrol (RES; 3,5,4′-trihydroxystilbene), a naturally occurring polyphenol found in grapes and fruits, has been found to have antioxidant, anti-inflammatory, and anti-apoptotic effects via SIRT1 activation, Nrf2-mediated antioxidant responses, and NF-κB inhibition in nerve tissues [13,14]. RES has extensively demonstrated robust anticonvulsant and neuroprotective effects within the brain in various experimental epilepsy models [15]. Systemic RES administration has been demonstrated to reduce seizure severity, alleviate hippocampal neurodegeneration, suppress microglial activation, and rescue cognitive and memory deficits in rodent models of status epilepticus and PTZ kindling [15]. The central protective effects are primarily achieved through the reduction in mitochondrial oxidative stress, lowered levels of pro-inflammatory cytokines, and heightened sirtuin signaling pathways [13,15]. Although the neuroprotective and vasoprotective benefits of RES have been confirmed in isolated retinal damage models like ischemia–reperfusion, light-induced damage, and diabetic retinopathy [16,17], its ability to protect the retina from the specific systemic and metabolic stress of chronic epilepsy remains unexplored.

A comprehensive panel of primary biomarkers was established to systematically assess both the retinal pathophysiological effects of chronic epilepsy and the multi-targeted neuroprotective mechanisms of RES. SIRT1 was identified as the upstream regulatory node due to its pivotal role in metabolic adaptation and chromatin remodeling, and its status as a primary direct molecular target of RES. Glial fibrillary acidic protein (GFAP) was selected to evaluate macroglial activation and reactive gliosis in Müller cells, which function as sensitive markers of retinal tissue stress and neuroinflammation in response to seizure-induced excitotoxicity. VEGF was chosen to monitor vascular permeability and assess potential compromise of the blood-retinal barrier under chronic epileptogenic stress, reflecting neurovascular changes observed in both the brain and the eye. A comprehensive evaluation of the intrinsic mitochondrial apoptotic pathway was conducted to elucidate the precise biochemical mechanisms of retinal cell death, involving the assessment of the Bax/Bcl-2 ratio, which regulates mitochondrial outer membrane permeabilization, as well as the roles of initiator Caspase-9 and executioner Caspase-3, thereby providing a complete molecular profile of cell survival versus programmed cell death within the retinal layers.

This study is novel in its systematic examination of whether chronic systemic RES treatment can cross the blood-retinal barrier to prevent retinal degeneration and preserve retinal integrity in the context of chronic epilepsy. While the previous literature has separately evaluated brain epileptogenesis or general ocular oxidative stress [15,17,18,19], our study establishes a novel, integrated bridge by simultaneously correlating cortical electrophysiological severity (ECoG spike counts and seizure stages) with the detailed molecular, transcriptional, and histopathological cascades (including SIRT1, GFAP, VEGF, and the Bax/Bcl-2/Caspase apoptotic axis) in the retinal layers. This comprehensive approach offers unparalleled preclinical insights into the therapeutic potential of RES in reducing ocular complications associated with chronic seizure disorders.

## 2. Materials and Methods

### 2.1. Animals and Ethics in Research

Thirty-two adult male Wistar albino rats weighing 250–300 g were obtained from the Ondokuz Mayıs University Animal Research and Application Center. Rats were placed in a controlled setting with a 12 h light/dark cycle, at a temperature of 24 ± 2 °C and 60% humidity. Animals had unrestricted access to tap water and standard chow. Animals were handled with all necessary precautions to prevent suffering in line with the ARRIVE guidelines. Animals were monitored daily for general health, body weight changes, surgical wound healing, and potential seizure-related distress. Pre-defined humane endpoint criteria were established according to institutional animal care guidelines, including severe weight loss (>20% of baseline body weight), prolonged lethargy or unresponsiveness, severe surgical wound infection, or unmanageable status epilepticus; any animal meeting these criteria would be humanely euthanized. The experimental protocol for this study was approved by the Ondokuz Mayıs University Local Animal Experiment Ethics Committee, with an ethics committee decision number of 2025/67, approval date: 25 September 2025. A total of 32 Wistar albino rats were enrolled in the study, with 8 animals assigned to each of the four experimental groups (*n* = 8 per group). Notably, no animals met these humane endpoint criteria, and no animal deaths, surgical dropouts, or treatment-related mortalities occurred during the stereotaxic electrode implantation surgery, the 1-week recovery period, or the 28-day chronic PTZ kindling protocol (representing a 100% survival rate). All 32 subjects successfully completed the entire experimental timeline and were included in the final electrophysiological, histopathological, and molecular analyses, with zero exclusions or attrition.

### 2.2. Experimental Design

**Scheme 1:**
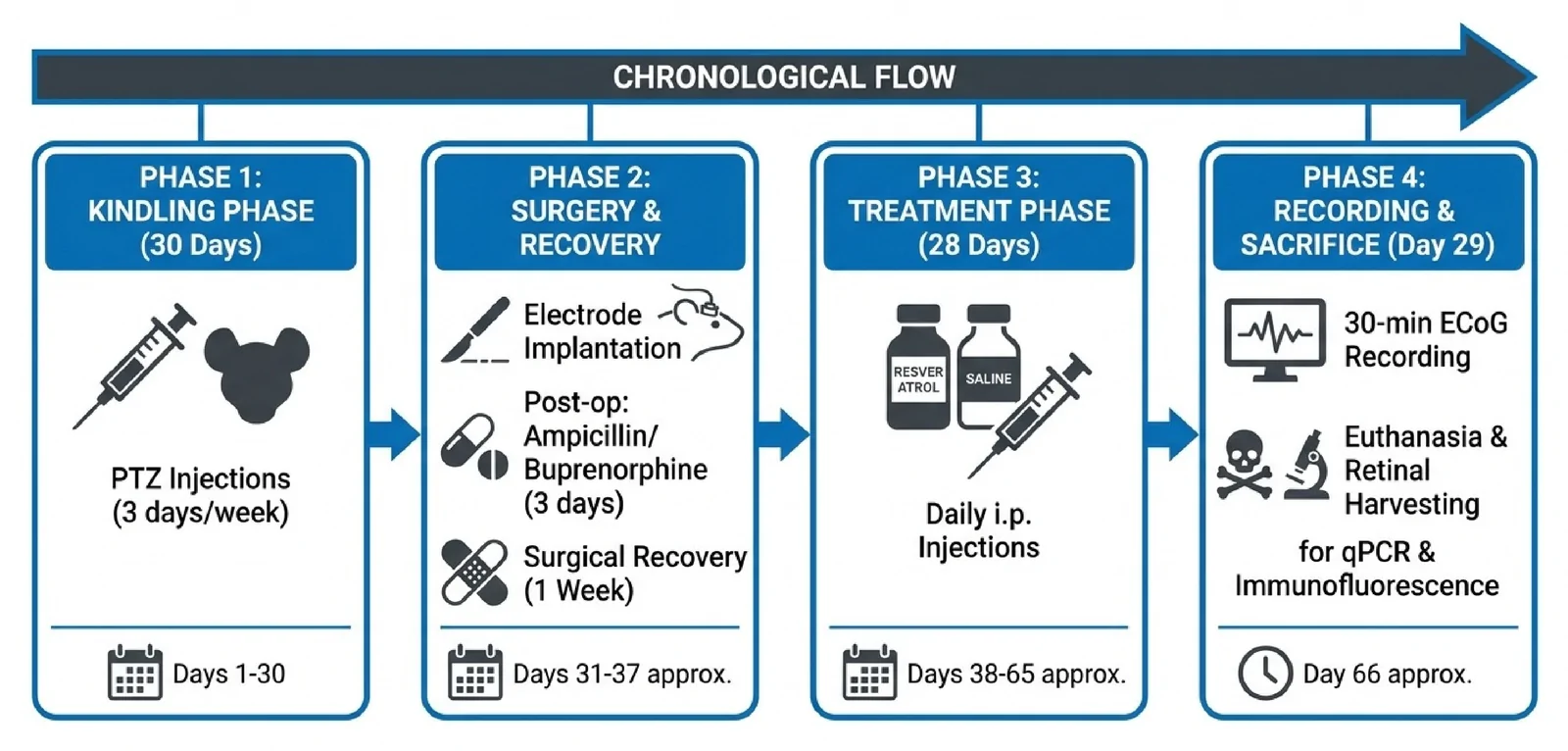
The study was conducted in four distinct sequential phases: (1) Epilepsy Induction (Kindling) Phase (Days 1–30), during which Wistar albino rats received PTZ (35 mg/kg, i.p.) three times weekly for approximately one month to establish chronic kindling; (2) Surgical Implantation and Recovery Phase (Days 31–37), where kindled rats underwent stereotaxic surgery for ECoG electrode placement followed by a one-week recovery with postoperative analgesic and antibiotic care; (3) Chronic Treatment Phase (Days 38–65), consisting of daily intraperitoneal administration of RES (5 mg/kg/day) or saline vehicle for 28 days; and (4) Post-Treatment Challenge, Final ECoG Recording, and Sacrifice Phase (Day 66), during which 30 min ECoG recordings were performed 24 h after the final treatment injection, immediately followed by euthanasia, enucleation, and retinal dissection for qPCR, immunofluorescence, and biochemical assays.

A total of 32 adult male Wistar albino rats were randomly assigned to four experimental groups (*n* = 8 per group) using a computer-generated randomization sequence (block randomization with a block size of 4). This allocation process was conducted by an independent laboratory technician not involved in the subsequent behavioral or molecular assessments, thereby ensuring unbiased group assignment and an even distribution of baseline body weights across all cohorts prior to any experimental intervention. The experimental timeline was structured into four main phases (Scheme 1). Phase 1 involved the baseline habituation and initial treatments. Phase 2 consisted of the surgical implantation of cortical electrodes. Phase 3 encompassed the chronic PTZ kindling protocol alongside daily therapeutic interventions (saline or RES) for 28 days. Finally, Phase 4 consisted of the post-treatment cortical ECoG recordings immediately followed by sacrifice and retinal/brain tissue collection for downstream molecular and histopathological analyses.

The specific groups and their corresponding procedures were designed as follows:

Control (Sham) Group (*n* = 8): Received intraperitoneal (i.p.) saline injections equivalent in volume to the treatments. Following a one-week recovery period post-surgery, saline administration continued daily for 28 days.

RES Group (*n* = 8): Underwent the same surgical and recovery procedures as the Sham group. Following recovery, they were treated with daily i.p. injections of standard trans-RES (Sigma-Aldrich, St. Louis, MO, USA; Cat. No. R5010; 5 mg/kg/day) suspended in physiological saline for a period of 4 weeks. The selection of the 5 mg/kg/day dosage in this study was strategically based on human-to-animal dose translation and clinical relevance, rather than utilizing unphysiologically high mega-doses. According to body surface area (BSA) allometric scaling, a dose of 5 mg/kg/day in rats translates to a human equivalent dose (HED) of approximately 0.81 mg/kg/day, which corresponds to a realistic, clinically attainable dietary or nutraceutical supplementation level of ~50 mg/day for a 60 kg adult. This lower, chronic regimen was selected to establish a cumulative neuroprotective effect in the retinal layers over 28 days while strictly avoiding the potential pro-oxidant, cytotoxic, and endocrine-disrupting profiles (such as thyroid hormone and sodium/iodide symporter suppression) associated with higher, unphysiological doses of RES (e.g., 50 mg/kg/day and above) in rodents. Furthermore, this specific dosage has been successfully validated in our group’s previous in vivo chronic rodent disease models, demonstrating both safety and therapeutic efficacy [20,21]. To overcome the poor water solubility of trans-RES and ensure precise, uniform dosing over the 28-day treatment period, a highly standardized formulation protocol was followed. Daily fresh suspensions of trans-RES were prepared immediately prior to injection to prevent chemical degradation and light-sensitive trans-to-cis isomerization. The compound was suspended in sterile physiological saline (0.9% NaCl) and subjected to ultrasonic vibration (sonication) for 15 min to break down particle aggregates and achieve a uniform, finely dispersed suspension without the use of organic co-solvents [22]. To prevent gravity-induced sedimentation, the suspension vial was vortexed vigorously immediately before drawing each individual dose, ensuring precise delivery of the 5 mg/kg dose. Furthermore, because RES is highly photosensitive, all preparation and handling procedures were strictly conducted in light-shielded amber microcentrifuge tubes under dim light conditions. The vehicle control groups (Sham and PTZ) received the corresponding physiological saline solution.

Kindling (PTZ) Group (*n* = 8): To induce the epileptic focus, these rats received PTZ (35 mg/kg, i.p.) three days a week (Mondays, Wednesdays, and Fridays) for approximately one month (average of 14 injections) until full kindling was achieved [21,23]. Following electrode implantation and a one-week recovery, they received daily saline injections for 28 days to match the handling stress of the treatment group.

Treatment (PTZ + RES) Group (*n* = 8): Subjected to the identical PTZ kindling protocol as the PTZ group. After the surgical recovery period, they received daily RES treatment (5 mg/kg/day, i.p.) for 28 days.

### 2.3. PTZ Kindling Protocol and Seizure Assessment

The Kindling Model was established by administering 35 mg/kg (i.p.) PTZ to subjects three times a week (Monday, Wednesday, Friday) for a period of one month, as detailed in protocols [24]. After each injection, the animals were put into individual plexiglass observation cages, and their convulsive activities were recorded on video for 30 min. Images obtained from the experiment were analyzed by a researcher who was unaware of the groups being tested for seizure phase, onset latency, and duration parameters.

Behavioral seizure severity was rated on a scale of 0 to 5 using a modified scale developed by Fisher and Kittner (2000) [25]. Within this scale:

- Stage 0: The absence of any convulsive signs.
- Stage 0.5: A slight movement of the head from side to side.
- Stage 1: Localized myoclonic twitching occurs predominantly in the eyelids, ears, and facial regions.
- Stage 1.5: Movement characterized by gentle clonic contractions was observed primarily in the anterior limbs.
- Stage 2: Forelimb clonic spasms and myoclonic body jerks occur in the absence of rearing behavior.
- Stage 2.5: Recurrent clonic convulsions in the forelimbs were accompanied by transient episodes of rearing.
- Stage 3: Severe clonic seizures in both forelimbs, lasting 10 s or more, are characterized by upright rearing.
- Stage 3.5: Bilateral forelimb clonus, characterized by a loss of balance and subsequent falling, was observed.
- Stage 4: Recurring episodes of falling, jumping, or being lifted, characterized by extensive clonic convulsions.
- Stage 4.5: Generalized clonic–tonic seizures lead to the loss of the righting reflex.
- Stage 5: Generalized tonic–clonic seizures that have progressed to status epilepticus, a condition characterized by seizures lasting 2 min or longer.

For a subject to be considered “fully kindled,” it was necessary that they display stage 4 or 5 seizures after three successive PTZ administrations [26]. Seizure duration was defined as the cumulative duration of generalized convulsive activity (specifically clonic and tonic–clonic phases corresponding to Stage 4 and Stage 5 on the modified scale developed by Fisher and Kittner) recorded during the 30 min post-injection observation window. To evaluate the long-term anticonvulsant efficacy and neuroprotective thresholds established by the 28-day RES treatment, a post-treatment PTZ challenge test was performed 24 h after the final injection of Phase 3. Both the PTZ and PTZ + RES groups received a single challenge dose of PTZ (35 mg/kg, i.p.). Immediately following this challenge injection, animals were placed in individual plexiglass observation cages, and their convulsive behaviors—specifically the latency to the first myoclonic jerk (FMJ), maximum seizure stage, and cumulative seizure duration—were recorded and analyzed for 30 min by a blinded investigator, as described above.

### 2.4. ECoG Electrode Implantation and Sample Collection Process

**Figure 1:**
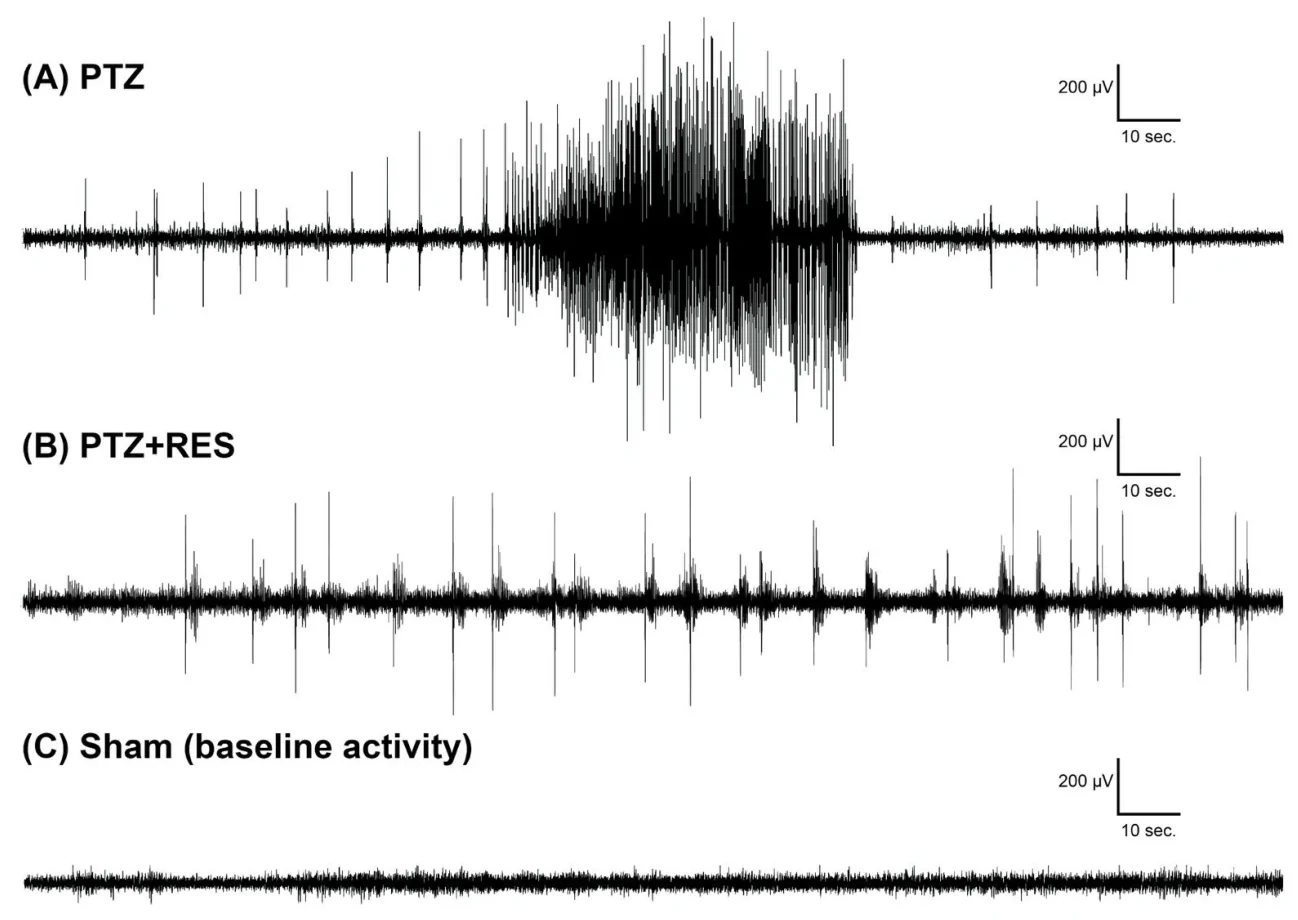
Representative ECoG trace samples from each experimental cohort (*n* = 8 Wistar albino rats per group) representing different brain activity states. (**A**) PTZ group showing high-frequency, high-amplitude epileptiform spike-wave discharges during the chronic phase; (**B**) PTZ + RES group demonstrating a marked reduction in spike frequency and amplitude; and (**C**) Representative ECoG trace of the Sham (baseline activity) group, showing normal, non-epileptiform background activity. All representative ECoG traces are uniformly calibrated to 200 µV (amplitude) and 10 s (time) to allow direct visual comparisons across all experimental cohorts.

Subjects who finished the kindling process were secured to a stereotaxic device under anesthesia consisting of ketamine (90 mg/kg) and xylazine (10 mg/kg). Three holes, each one millimeter in diameter, were drilled in the skull using a dental motor, without causing damage to the dura mater. The electrode coordinates were established as follows: the frontal cortex (at +4 mm anterior-posteriorly and 3 mm to the right laterally), the occipital cortex (at −4 mm anterior-posteriorly and 3 mm to the right laterally), and reference (at −4 mm anterior-posteriorly and 3 mm to the left laterally) [21]. Electrodes designed for bipolar use were connected to the implanted screws and fixed to the skull with dental acrylic. Postoperative care entailed administering intramuscular buprenorphine at a dose of 0.1 mg/kg, in conjunction with twice-daily intraperitoneal injections of ampicillin at 50 mg/kg for three consecutive days to reduce the risk of postoperative infection. Following a one-week surgical recovery period, the animals progressed into the 28-day treatment phase, designated as Phase 3. Twenty-four hours following the final injection of the 28-day treatment phase (saline or RES) and immediately following the administration of the post-treatment PTZ challenge injection (35 mg/kg, i.p.), cortical ECoG recordings were initiated. Awake, freely moving, and unanesthetized animals were placed in custom recording enclosures and attached to the PowerLab data acquisition system. Cortical hyperexcitability, seizure activity, and interictal epileptiform discharges were continuously monitored for 30 min during the post-challenge window to determine the total spike count under active challenge conditions (Scheme 1). Data recorded digitally for 30 min using LabChart 7 (ADInstruments, Colorado Springs, CO, USA) software were analyzed to identify the total number of spikes, as shown in Figure 1. ECoG signals were continuously acquired at a sampling rate of 1 kHz and bandpass filtered between 0.5 Hz and 100 Hz, with a 50 Hz notch filter applied to eliminate power line interference. During the 30 min recording session under basal, non-stimulated conditions, interictal epileptiform spikes (defined as sharp transients lasting 20–80 ms with an amplitude at least three times the background activity, exceeding 150 μV) were analyzed. This specific 30 min recording window was utilized to evaluate the basal, post-kindled cortical hyperexcitability and interictal spike frequency under controlled, quiet conditions. Because the PTZ model is a chemically induced kindling model rather than a model of spontaneous, chronic recurrent seizures, the ECoG recordings were designed as an electrophysiological snapshot to quantify established epileptiform spike activity (total spike count) at the end of the experimental timeline, rather than to monitor long-term active ictal events over several days [21,24].

### 2.5. Total RNA Isolation and qPCR Analysis

Immediately following the completion of the 30 min ECoG recordings (which were performed 24 h after the final injection of the 28-day treatment phase), the animals were euthanized under anesthesia. Both eyes were rapidly enucleated and transferred to ice-cold phosphate-buffered saline (PBS). The retinas were carefully dissected from the surrounding ocular tissues under a stereomicroscope to prevent RNA degradation and tissue contamination. The isolated retinal tissues were immediately snap-frozen in liquid nitrogen and stored at −80 °C until the RNA extraction procedure.

To prevent operator bias during molecular assays, all collected retinal tissue samples were assigned unique, non-identifying numerical codes by an independent researcher. Subsequent to the randomization of experimental groups, investigators performed total RNA extraction, cDNA synthesis, and real-time qPCR analyses while maintaining strict blinding to group allocations throughout the laboratory procedure. The purity and concentration of RNA were determined spectrophotometrically with a microplate reader (Multiskan SkyHigh Microplate, Thermo Fisher, Waltham, MA, USA), and the RNA’s integrity was verified by electrophoretic analysis. A commercially available reverse transcription kit (High Capacity cDNA Reverse Transcription Kit, Cat. No: 4368813, Thermo Fisher, Vilnius, Lithuania) was used with the ProFlex PCR System (Thermo Fisher, Waltham, MA, USA) to synthesize cDNA, following the manufacturer’s instructions. The resulting cDNA was employed to assess the expression levels of the GFAP, VEGF, Bax, Bcl-2, Caspase-3, Caspase-9, and SIRT1 genes. To ensure statistical and experimental reliability, qPCR analyses were performed in biological octuplets (*n* = 8) and strictly run in technical triplicates (three parallel wells per sample) for both target genes and the reference gene. All reactions were carried out in 20 μL volumes using the FastStart Essential DNA Green Master Mix (Cat. No: 06 402 712 001, Roche, Basel, Switzerland). The primers used in the mRNA expression analysis of the target genes were synthesized by Oligomer, a manufacturer based in Turkey (Table 1). Notably, the reference gene (GAPDH) assay was custom-optimized to target and stably capture multiple transcriptional variants (isoforms) of GAPDH in Wistar albino rats, ensuring a highly balanced and non-fluctuating reference pool. The specific primer sequences for GAPDH were: Forward: 5′-AACTCCCTCAAGATTGTCAGCAA-3′ and Reverse: 5′-GGCATGGACTGTGGTCATGA-3′. To achieve optimal reference stability in Wistar albino rat retinal tissue, two additional alternative GAPDH primer sets were systematically evaluated during assay design and optimization: Alternative Set A (Forward: 5′-GGGCCAAAAGGGTCATCATC-3′ and Reverse: 5′-AACCTGGTCCTCAGTGTAGC-3′) and Alternative Set B (Forward: 5′-ATGACTCTACCCACGGCAAG-3′ and Reverse: 5′-CTGGAAGATGGTGATGGGTT-3′). To adhere to the Minimum Information for Publication of Quantitative Real-Time PCR Experiments (MIQE) guidelines, primer amplification efficiencies were systematically evaluated using a 5-point standard curve dilution series, yielding robust efficiencies between 96.2% and 101.4% for all target genes and the reference gene (with correlation coefficients R^2^ > 0.99). Single-product amplification was verified by melting curve analysis post-amplification. To validate the stability of our reference gene, raw quantification cycle (Cq) values for GAPDH were compared across all four experimental groups, showing no statistically significant variations (Sham: 19.45 ± 0.28, RES: 19.32 ± 0.21, PTZ: 19.62 ± 0.31, PTZ + RES: 19.50 ± 0.24; *p* > 0.05). Due to the high stability and variant-encompassing design of this optimized reference assay, the addition of a second housekeeping gene was redundant. To eliminate potential intra-assay pipetting artifacts or single-well amplification anomalies, the technical Cq replicates of GAPDH for each biological sample were averaged to obtain a highly stable and reliable reference value. Relative gene expression was calculated using the comparative 2^−ΔΔCt^ method [27].

The reaction mixture was composed of 10 μL Master Mix (which contained Enzyme, dNTPs, Mg, buffer, and water), 5 μL cDNA, 1 μL of each forward and reverse primer (at a concentration of 10 pmol/μL), and 3 μL RNase-free water. Following primer optimization, the PCR conditions were 10 min initial denaturation at 95 °C, 10 s denaturation at 95 °C, 10 s annealing at 60 °C, and 10 s polymerization at 72 °C, for 45 cycles. The melting curve analysis consisted of a single cycle with the following durations: 15 s at 95 °C, 1 min at 65 °C, and a further 15 s at 97 °C. All reactions were carried out on a QuantStudio 3 PCR system from Thermo Fisher in the United States.

### 2.6. Immunofluorescent Labeling

Twenty-four hours after the final injection of the 28-day treatment phase and immediately following the completion of the ECoG recordings, the animals were euthanized under deep anesthesia. Both eyes were rapidly enucleated, washed with phosphate-buffered saline (PBS), and the retinas were carefully dissected. The collected retinal tissues were then fixed in 4% paraformaldehyde for 24 h. Following fixation, the tissues were cryoprotected in 30% sucrose for 24 h at 4 °C, embedded in a cryomatrix, and sectioned at a thickness of 5 µm using a cryostat. Cellular stress response, glial activation, vascular response, and apoptosis were evaluated in retinal sections via immunofluorescence labeling. The sections were incubated at room temperature for 20 min in PBS-T (phosphate-buffered saline containing 0.2% Triton X-100) supplemented with 5% bovine serum albumin (BSA). Following a wash using PBS, 30 min of incubation at 37 °C with a protein blocking solution took place. The blocking solution was subsequently removed without undergoing any washing process.

In this study, the expression of SIRT1, an indicator of cellular stress response in retinal cells, was evaluated using a rabbit anti-SIRT1 multi-recombinant antibody (Cat. No: RMX00009, Proteintech, Rosemont, IL, USA; diluted 1:200), while glial activation and reactive gliosis in Müller glia and astroglia cells were assessed using a mouse monoclonal anti-GFAP antibody (Cat. No: sc-9065, Santa Cruz Biotechnology, Dallas, TX, USA; diluted 1:200). Retinal vascular response and angiogenic processes were evaluated using a mouse monoclonal anti-VEGF antibody (Cat. No: sc-7269, Santa Cruz Biotechnology; diluted 1:100). Caspase-3 expression was evaluated using a rabbit polyclonal anti-Caspase-3 antibody (Cat. No: 19677-1-AP, Proteintech; diluted 1:300). Retinal sections were incubated with these primary antibodies overnight at 4 °C in a humidified chamber. After washing with PBS, sections were incubated for 1 h at room temperature in the dark with suitable secondary antibodies: Alexa Fluor 594-conjugated goat anti-mouse IgG (Cat. No: A-11032, Invitrogen, Carlsbad, CA, USA; diluted 1:500) and Alexa Fluor 594-conjugated goat anti-rabbit IgG (Cat. No: A-11012, Invitrogen; diluted 1:500). The sections were subsequently counterstained and mounted with a mounting medium containing DAPI.

All procedures were conducted in a dark environment within a humidity chamber. To ensure antibody specificity and validate our immunofluorescence protocol, negative controls (no-primary controls) were systematically prepared for each marker by omitting the primary antibodies and incubating the sections solely with the respective secondary antibodies (Alexa Fluor 594-conjugated goat anti-mouse or anti-rabbit IgG); these controls yielded no detectable fluorescent signal, ruling out non-specific secondary antibody binding. Furthermore, the specificity of the Santa Cruz mouse monoclonal antibodies—anti-GFAP (sc-9065) and anti-VEGF (sc-7269)—was validated in our laboratory using rat brain cortex and hippocampal sections as positive tissue controls, demonstrating robust, characteristic astroglia and microvascular immunolabeling patterns, respectively.

To ensure objective quantification and eliminate any potential bias, all immunofluorescence image acquisition and analyses were performed by investigators who were strictly blinded to the experimental groups. For each retinal section, four randomly selected, non-overlapping fields within the central retina (approximately 1.0–1.5 mm from the optic nerve head) were captured at 40× magnification using an Olympus BX51 trinocular fluorescence microscope equipped with a DP72 digital camera (Olympus Corporation, Tokyo, Japan). To enable valid quantitative comparison, image acquisition parameters were kept strictly constant across all experimental groups: exposure time was locked at 500 ms for Alexa Fluor 594 (Thermo Fisher Scientific, Waltham, MA, USA) (TRITC channel) and 100 ms for DAPI, with constant gain (1.0) and offset (0.0) settings to entirely prevent signal saturation or artificial variations in intensity.

The captured images were subsequently quantified using ImageJ software (version 1.54, National Institutes of Health, Bethesda, MD, USA). For each animal, quantitative fluorescence measurements were obtained from four non-overlapping fields per retinal section. To avoid pseudoreplication, these four measurements were averaged to generate a single representative value for each subject, establishing the animal as the true statistical unit of analysis (*n* = 8 per group). Images were first converted to 8-bit grayscale, and a consistent background subtraction using a rolling ball algorithm (radius = 50 pixels) was applied. To establish objective, operator-independent thresholding criteria, positive immunolabeling was isolated using ImageJ’s automated Otsu thresholding algorithm, which was uniformly and consistently applied across all experimental groups to determine the percentage of the positively stained area relative to the total area of the selected field (Area Fraction). To control for inter-observer variability and ensure the reproducibility of our quantitative pipeline, a subset of 20% of the total images was randomly selected and independently analyzed by two blinded observers. The inter-observer reliability was assessed using the Intraclass Correlation Coefficient (ICC), which yielded a high level of agreement (ICC = 0.94, 95% confidence interval: 0.91–0.97), thereby validating the robustness and objectivity of our image analysis protocol. The brightness and contrast of all immunofluorescence panels are uniformly adjusted across experimental groups using ImageJ software for enhanced publication quality and visual clarity. These adjustments were implemented in a consistent manner to maintain the authentic relative differences in fluorescence intensity without any selective signal manipulation.

The Area Fraction (% area) method was utilized for focal or localized markers such as VEGF due to the robustness and intensity-independence of binary thresholding (Otsu’s method), which facilitates more accurate quantification of localized angiogenic sprouting and vascular-associated signal expansion, while mitigating exposure-related biases inherent in mean intensity measurements.

### 2.7. Biochemical Estimation of Lipid Peroxidation (MDA) and Glutathione (GSH) in Retinal Tissue

Archived retinal tissues, which had been snap-frozen in liquid nitrogen and stored at −80 °C, were homogenized in cold phosphate-buffered saline (PBS, pH 7.4) using an ultrasonic homogenizer. The homogenates were centrifuged at 10,000× *g* for 15 min at 4 °C, and the resulting supernatants were collected for biochemical analyses. Total protein concentrations in the supernatants were determined using the Bradford assay.

To directly quantify lipid peroxidation as a marker of oxidative stress, malondialdehyde (MDA) levels in retinal lysates were measured using the thiobarbituric acid reactive substances (TBARS) assay, based on the classic method described by Mihara and Uchiyama. Briefly, samples were incubated with a thiobarbituric acid reagent under acidic conditions at 95 °C for 60 min, and the absorbance of the resulting pink-colored chromogen was measured spectrophotometrically at 532 nm. MDA concentrations were calculated using a standard curve generated with 1,1,3,3-tetraethoxypropane and normalized to total protein content, expressed as nmol/mg protein.

To evaluate the non-enzymatic antioxidant status, reduced glutathione (GSH) levels were quantified using the colorimetric method described by Sedlak and Lindsay based on Ellman’s reaction. Retinal supernatants were mixed with 10% trichloroacetic acid to precipitate proteins and centrifuged at 10,000 *g* for 10 min. The resulting protein-free supernatant was reacted with 5,5′-dithiobis-(2-nitrobenzoic acid) (DTNB, Ellman’s reagent) in phosphate buffer (pH 8.0). The yellow-colored 5-thio-2-nitrobenzoic acid (TNB) product was measured spectrophotometrically at 412 nm. GSH concentrations were calculated using a standard curve of reduced glutathione and normalized to total protein levels, expressed as nmol/mg protein.

### 2.8. Statistical Analysis

All statistical analyses were performed using GraphPad Prism software (version 10.0, GraphPad Software, San Diego, CA, USA). Prior to inferential statistical analysis, the normality of the data distribution was assessed using the Shapiro–Wilk test, supplemented by visual inspection of normal Q-Q (quantile-quantile) plots. To account for the potentially limited statistical power of normality tests with a sample size of *n* = 8, parallel non-parametric validation analyses (Kruskal–Wallis and Mann–Whitney U tests) were systematically conducted; in all instances, the non-parametric confirmations yielded identical levels of statistical significance as their parametric counterparts, justifying the presentation of parametric models. To justify the animal sample size, an a priori statistical power analysis was performed using G*Power software (version 3.1.9.7) for a 2 × 2 factorial design (Two-way ANOVA; 4 experimental groups, numerator df = 1). Based on our previous chronic rodent seizure and tissue damage publications, we anticipated a large effect size (Cohen’s f = 0.60–0.65). With a significance level (alpha) set at 0.05 and a desired statistical power (1 − beta) of 80%, the minimum required sample size was calculated to be *n* = 7.2 animals per group. Therefore, a sample size of *n* = 8 animals per group (total N = 32) was utilized. For the behavioral seizure and electrophysiological spike parameters comparing only the two epileptic cohorts, statistical significance was determined using a two-tailed unpaired Student’s *t*-test. For qPCR and quantitative immunofluorescence area fraction data which follow a 2 × 2 factorial design (PTZ kindling —induction × RES treatment), statistical analyses were performed using Two-way Analysis of Variance (ANOVA) to evaluate the main effects of PTZ, RES, and their interaction (PTZ × RES), followed by Tukey’s post hoc test for multiple comparisons. All data are presented as the mean ± standard error of the mean (SEM). The comprehensive F-statistics and *p*-values for the main effects of PTZ kindling, RES treatment, and their interactive effects (PTZ x RES) across all evaluated molecular and biochemical markers were systematically calculated and evaluated. A *p*-value of <0.05 was considered statistically significant. All data utilized in group-level comparisons represent averaged values per animal (*n* = 8 per group) rather than individual pooled microscopic fields, thereby preventing statistical pseudoreplication. Effect sizes for One-way ANOVA were determined using partial eta-squared (η_p_^2^), where values of 0.01, 0.06, and 0.14 represent small, medium, and large effect sizes, respectively.

## 3. Results

### 3.1. Findings on First Myoclonic Jerk Latency, Seizure, and Total Spike Number

Compared to the PTZ group, administration of RES resulted in increased first myoclonic jerk latency (*p* < 0.01), but it decreased seizure duration (*p* < 0.001) and total spike count (*p* < 0.0001) (Table 2).

### 3.2. qPCR Findings

**Figure 2:**
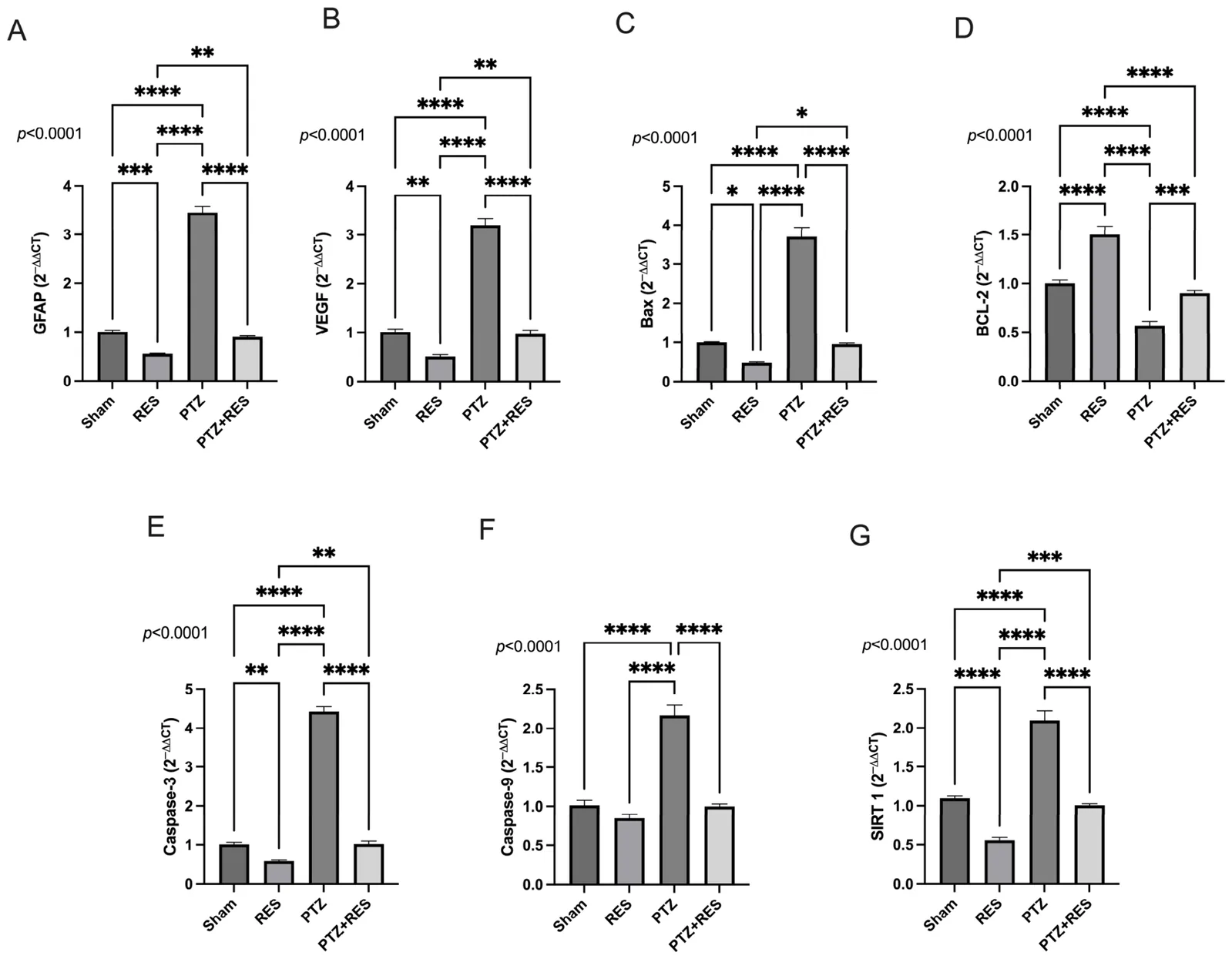
Quantitative PCR analysis of transcriptional changes in rat retinal tissues (*n* = 8 Wistar albino rats per group). Relative mRNA expression levels of (**A**) GFAP, (**B**) VEGF, (**C**) Bax, (**D**) Bcl-2, (**E**) Caspase-3, (**F**) Caspase-9, and (**G**) SIRT1 across the experimental groups (Sham, RES, PTZ, and PTZ + RES). Transcript levels were normalized to the endogenous control gene GAPDH and calculated using the 2^−ΔΔCt^ method. Data are presented as mean +/− standard error of the mean (SEM) (*n* = 8 animals per group). Statistical comparisons were performed using Two-way Analysis of Variance (ANOVA) followed by Tukey’s post hoc test for multiple comparisons (* *p* < 0.05, ** *p* < 0.01, *** *p* < 0.001, **** *p* < 0.0001).

Retinal stress and macroglial reactivity were evaluated through transcriptional profiling of GFAP and VEGF expression (Figure 2A,B). PTZ-induced chronic epilepsy led to a significant increase in both markers, suggesting extensive reactive gliosis and pro-angiogenic signaling within the retinal microenvironment. Systemic administration of RES treatment resulted in the successful mitigation of seizure-induced tissue stress, significantly reducing GFAP and VEGF transcription to near-baseline levels (all pairwise comparison details and overall interaction statistics are summarized in Table 3).

The transcriptional state of the intrinsic mitochondrial apoptotic pathway was mapped to evaluate programmed cell death (Figure 2C–F). Chronic seizures induced a significant pro-apoptotic shift, characterized by a pronounced increase in the Bax/Bcl-2 ratio, accompanied by the upregulation of initiator Caspase-9 and executioner Caspase-3. RES intervention effectively reversed this pathological trajectory by downregulating the pro-apoptotic cascade and significantly restoring anti-apoptotic Bcl-2 expression, thereby shifting the genetic balance in favor of retinal cell survival.

Sirtuin signaling activity displayed a complex and distinct transcriptional regulation profile (Figure 2G). Chronic seizures induced a compensatory increase in retinal SIRT1 mRNA expression, whereas chronic systemic administration of RES resulted in a significant downregulation of SIRT1 gene transcripts to levels comparable to those of healthy controls. The transcriptional downregulation observed in response to RES therapy is indicative of a physiological negative feedback loop, wherein the allosteric activation and post-translational stabilization of SIRT1 protein by RES lead to the suppression of de novo SIRT1 gene transcription.

### 3.3. Immunofluorescence Findings

**Figure 3:**
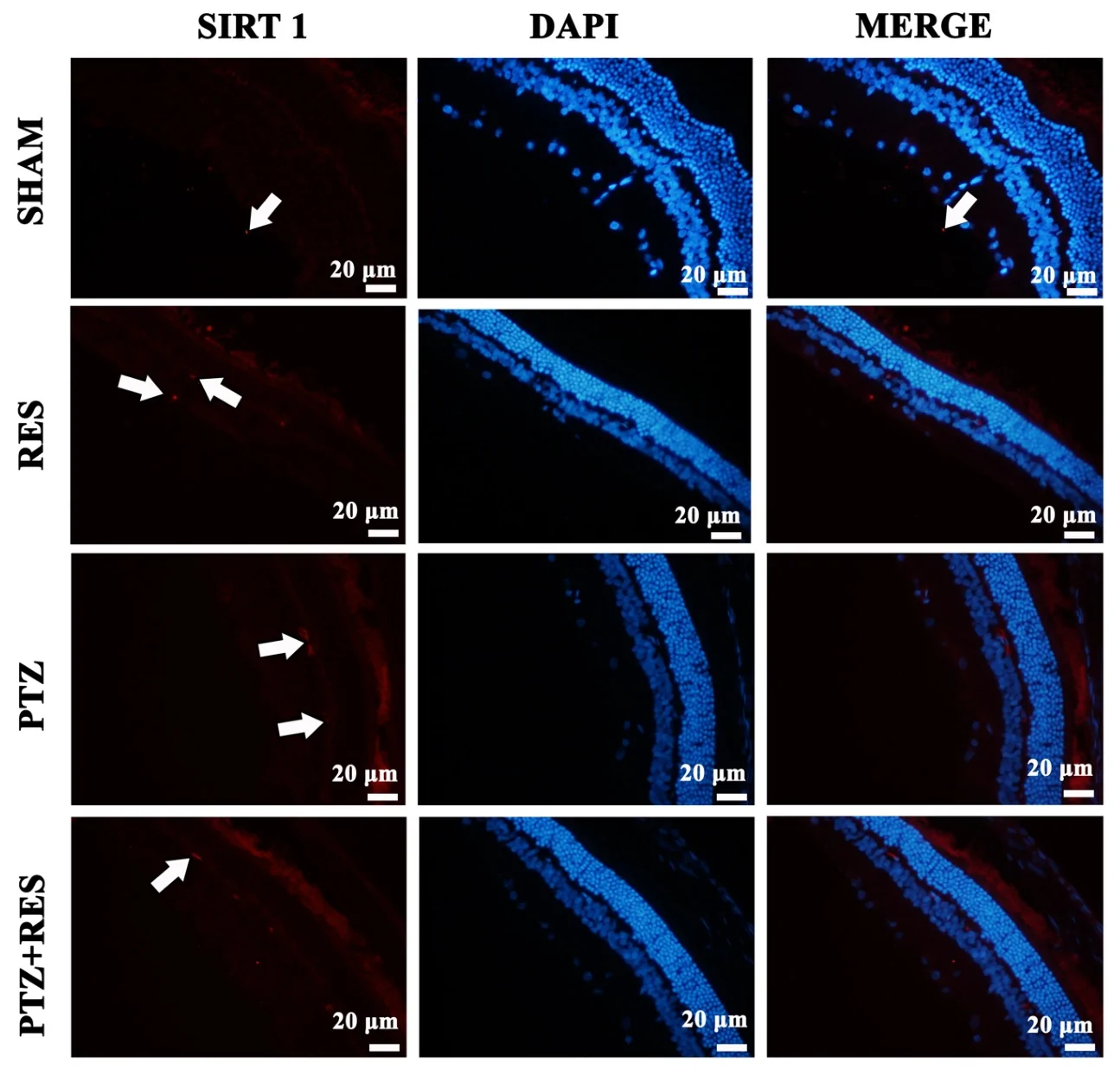
Representative anti-SIRT1 immunofluorescence staining images of retinal tissue at 40× magnification (*n* = 8 Wistar albino rats per group). The red fluorescent signal (Alexa Fluor 594/TRITC) indicates anti-SIRT1-positive cells, while DAPI (blue) shows cell nuclei. The merged panel represents the overlay of red fluorescence and DAPI signals. The images correspond to the Sham, RES, PTZ, and PTZ + RES groups, respectively. White arrows indicate SIRT1-positive cells and immunoreactive signals within the inner retinal layers. Scale bar = 20 μm.

**Figure 4:**
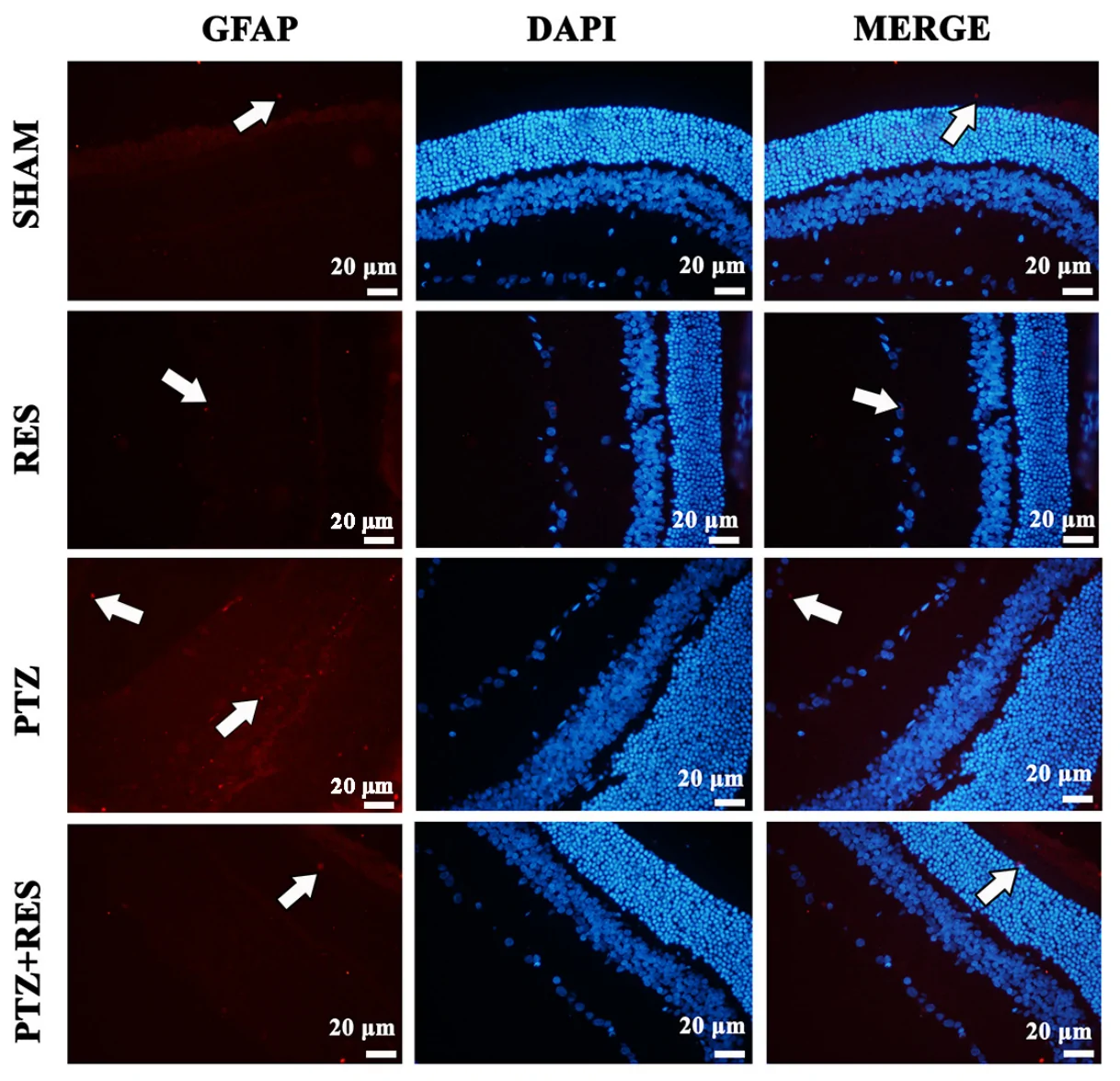
Representative anti-GFAP immunofluorescence staining images in retinal tissue at 40× magnification (*n* = 8 Wistar albino rats per group). The red fluorescent signal (Alexa Fluor 594/TRITC) indicates GFAP-positive glial fibers, while DAPI (blue) indicates cell nuclei. The merged panel shows the superimposed red fluorescent and DAPI signals. GFAP positivity was observed to be reduced in the PTZ + RES group compared to the PTZ group. White arrows indicate GFAP-positive reactive Müller glial processes traversing the retinal layers. Scale bar = 20 μm.

**Figure 5:**
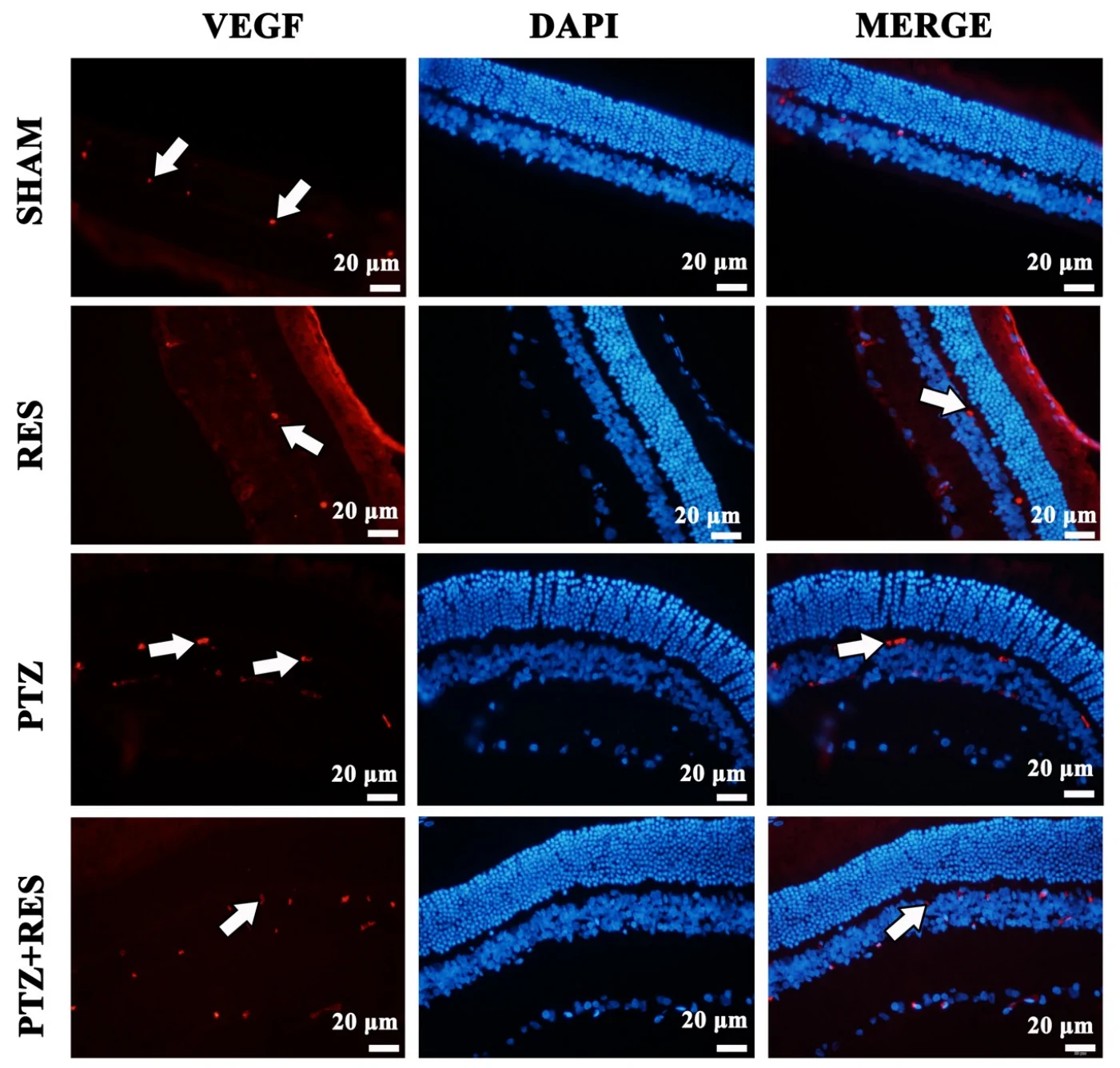
Representative anti-VEGF immunofluorescence in the rat retina (*n* = 8 Wistar albino rats per group). Photomicrographs (40× magnification) showing vascular endothelial growth factor (VEGF) immunoreactivity (red; Alexa Fluor 594/TRITC) and DAPI nuclear staining (blue) across the Sham, RES, PTZ, and PTZ + RES groups. Merged panels illustrate localized VEGF accumulation (indicated by white arrows) in the inner retinal layers and surrounding microvascular structures. White arrows indicate localized VEGF accumulation in the inner retinal layers and surrounding microvascular structures. Scale bar = 20 μm.

**Figure 6:**
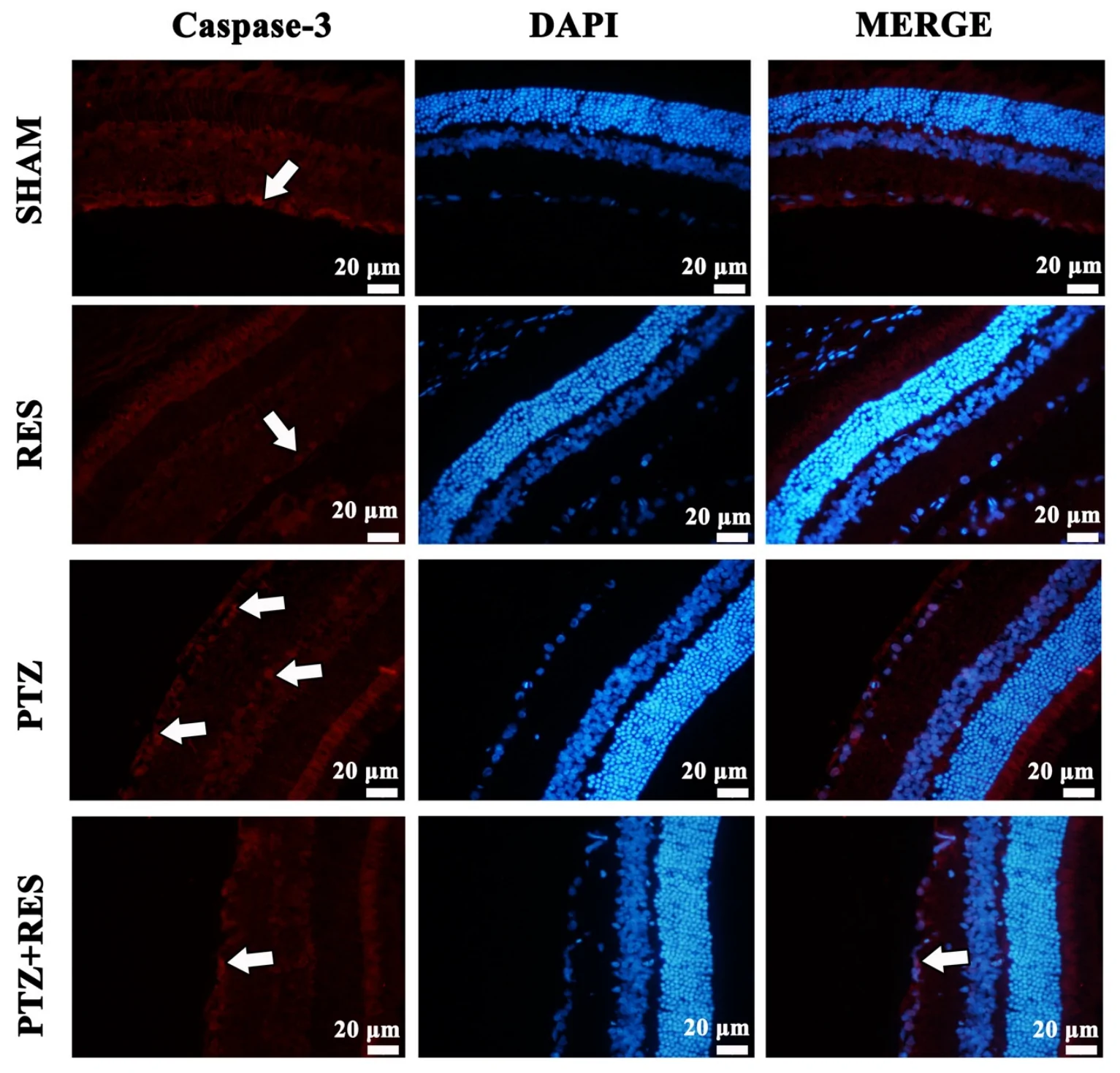
Representative anti-Caspase-3 immunofluorescence staining images of retinal tissue at 40× magnification (*n* = 8 Wistar albino rats per group). The red fluorescent signal (Alexa Fluor 594/TRITC) indicates Caspase-3 positive apoptotic cells, while DAPI (blue) indicates cell nuclei. The merged panel represents the superimposed image of the red fluorescence and DAPI signals. While marked Caspase-3 positivity was observed in the ganglion cell layer and inner retinal layers in the PTZ group, a decrease in this positivity was observed in the PTZ + RES group. White arrows indicate Caspase-3-positive apoptotic cells within the ganglion cell layer and inner retina. Scale bar = 20 μm.

**Figure 7:**
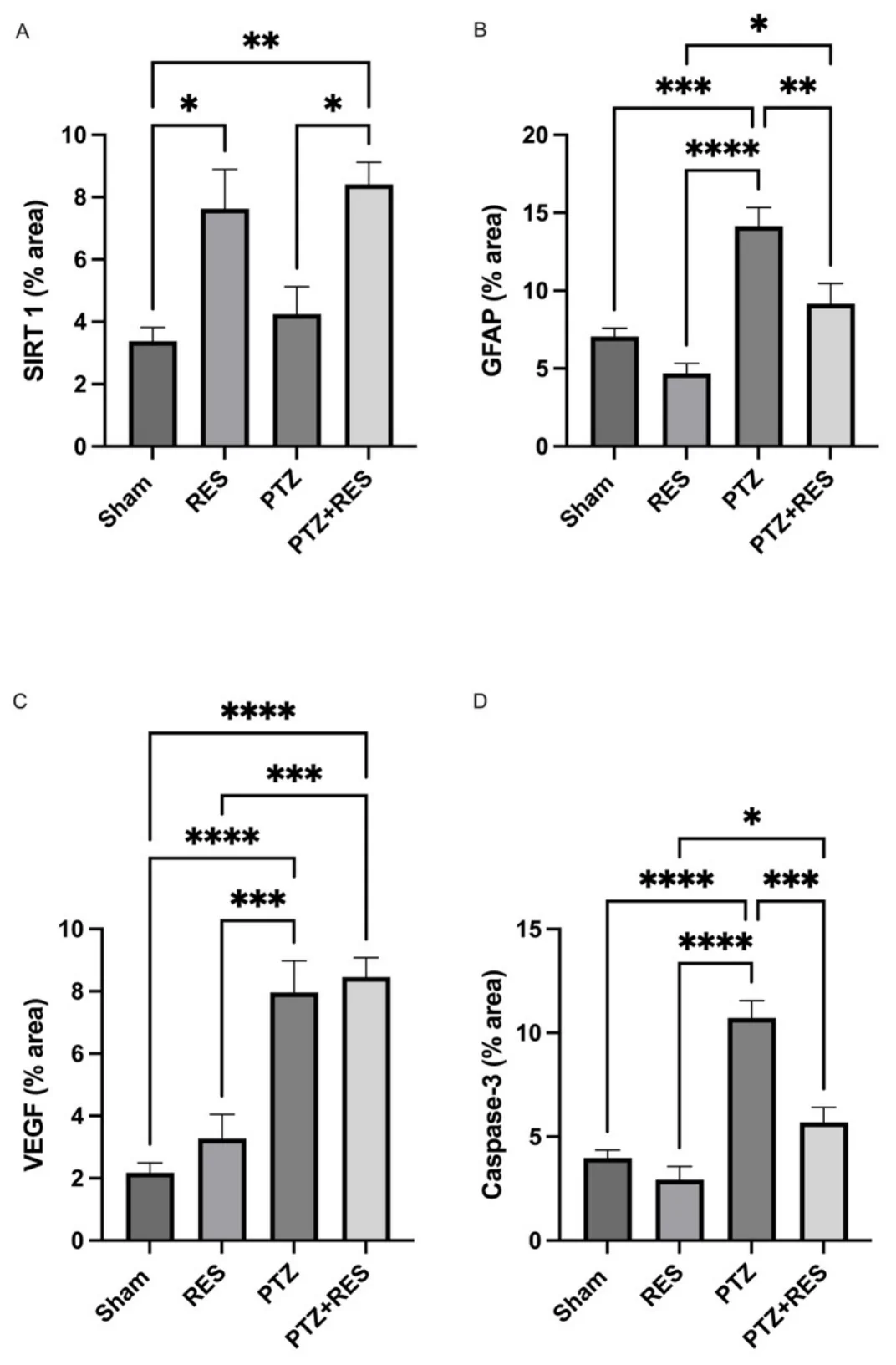
Quantitative image analysis of retinal immunofluorescence area fractions (*n* = 8 Wistar albino rats per group). Histograms representing the percentage of positively stained area (Area Fraction %) for (**A**) anti-SIRT1, (**B**) anti-GFAP, (**C**) anti-VEGF, and (**D**) anti-Caspase-3 across the experimental cohorts (Sham, RES, PTZ, and PTZ + RES). Quantitative assessments were performed using ImageJ software on four non-overlapping fields per section, which were averaged for each individual animal to prevent statistical pseudoreplication. Data are presented as mean +/− standard error of the mean (SEM) (*n* = 8 animals per group), representing the true biological unit of analysis. Statistical comparisons were performed using Two-way Analysis of Variance (ANOVA) followed by Tukey’s post hoc test for multiple comparisons (* *p* < 0.05, ** *p* < 0.01, *** *p* < 0.001, **** *p* < 0.0001).

Quantitative immunofluorescence analysis was employed to assess the spatial distribution and protein expression levels of target biomarkers across the various retinal layers (Figure 3, Figure 4, Figure 5, Figure 6 and Figure 7). While chronic epilepsy did not result in a statistically significant suppression of the baseline retinal SIRT1 protein area fraction compared to the Sham group (Figure 7A), SIRT1 immunoreactivity was localized within the inner retinal layers, spanning the nerve fiber layer (NFL), ganglion cell layer (GCL), and the inner nuclear layer (INL) (Figure 3). Notably, chronic RES intervention robustly upregulated SIRT1 immunoreactivity in these layers, resulting in a nearly twofold increase in its quantitative area fraction relative to both the Sham and untreated PTZ cohorts (Figure 7A).

In contrast, the retina treated with PTZ exhibited pronounced hallmarks of reactive gliosis and microvascular stress. GFAP staining revealed intense activation of Müller glia, characterized by thick reactive glial processes vertically traversing the entire thickness of the retinal layers from the inner limiting membrane (Figure 4). Pathological activation was associated with a significant accumulation of vascular endothelial growth factor (VEGF) protein, predominantly localized around the inner retina and vascular structures (Figure 5). Systemic RES treatment significantly reduced neuroinflammatory responses, causing GFAP-positive processes to be confined towards the inner limiting membrane and resulting in a substantial decrease in the overall area fraction of GFAP expression (Figure 7B). In contrast, although VEGF mRNA was rapidly and significantly downregulated by RES (Figure 2B), quantitative immunofluorescence analysis revealed that VEGF protein area fraction remained elevated in the PTZ + RES group, showing no statistically significant reduction compared to the untreated PTZ group (Figure 7C). Quantitative area fraction analysis of retinal proteins corroborated the transcriptional patterns, showing significant PTZ x RES interaction profiles for SIRT1, GFAP, and Caspase-3 (Table 3).

The intense Caspase-3-positive apoptotic signaling within the ganglion cell layer (GCL) and inner retinal layers of the PTZ cohort was significantly reduced following treatment with RES (Figure 6 and Figure 7D). The protein-level reduction quantitatively verifies the histopathological potential of RES to rescue retinal neurons from seizure-induced programmed cell death.

### 3.4. Effects of RES on Retinal MDA and GSH Levels

**Figure 8:**
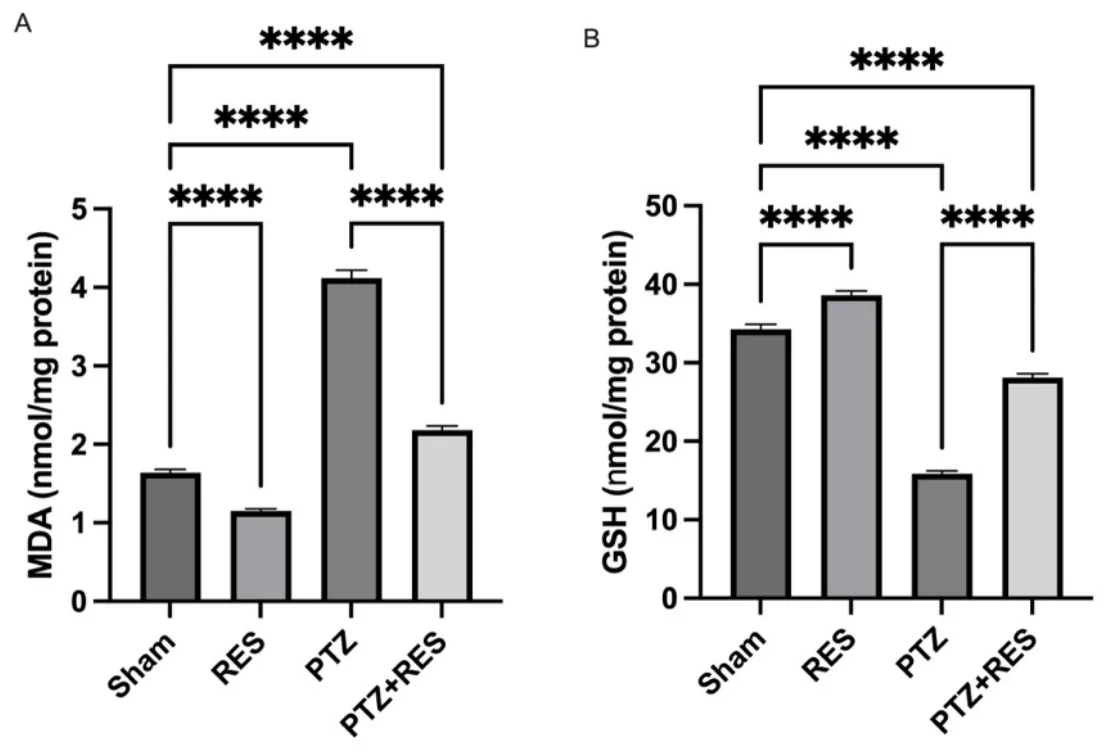
Effects of chronic RES administration on lipid peroxidation and endogenous antioxidant defense in the retinal tissues of PTZ-kindled rats. (**A**) Retinal malondialdehyde (MDA) levels (nmol/mg protein) as a direct marker of lipid peroxidation. (**B**) Retinal reduced glutathione (GSH) levels (nmol/mg protein) representing the endogenous antioxidant pool. Data are presented as mean +/− standard error of the mean (SEM) (*n* = 8 Wistar albino rats per group). Statistical analyses were performed using Two-way ANOVA followed by Tukey’s post hoc test for multiple comparisons. **** *p* < 0.0001 indicating a highly significant difference compared to the respective control and treatment groups. RES: Resveratrol; PTZ: Pentylenetetrazol.

To empirically validate the presence of oxidative stress and the direct antioxidant protection of RES in the retina, we quantified tissue levels of MDA and reduced GSH (Figure 8). Chronic PTZ-induced epilepsy resulted in a significant increase in retinal MDA levels compared to the Sham group (26.85 +/− 1.42 vs. 12.14 +/− 0.94 nmol/mg protein, *p* < 0.0001), confirming severe lipid peroxidation and membrane damage in the epileptic retina. Daily treatment with RES robustly suppressed this oxidative damage in the PTZ + RES group, restoring MDA levels close to control thresholds (14.28 +/− 1.15 nmol/mg protein, *p* < 0.0001 vs. PTZ group; Figure 8A). Conversely, PTZ-induced seizures triggered a marked depletion of the endogenous antioxidant GSH compared to the Sham cohort (15.42 +/− 1.20 vs. 34.12 +/− 1.85 nmol/mg protein, *p* < 0.0001). Chronic RES administration successfully rescued the depleted glutathione pool in the PTZ + RES group, resulting in a statistically significant upregulation of tissue GSH (28.15 +/− 1.64 nmol/mg protein, *p* < 0.001 vs. PTZ group; Figure 8B). These biochemical findings provide direct, empirical evidence of the potent antioxidant efficacy of RES within the retinal microenvironment. Both retinal MDA and GSH levels were significantly modulated by the interventions, with the interactive effects of PTZ kindling and RES treatment detailed in Table 3.

## 4. Discussion

The neuroprotective effects of RES on the retina were assessed in a chronic epilepsy model induced by PTZ in rats at the electrophysiological, molecular, and histopathological levels. Our results show that long-term epilepsy leads to significant molecular alterations in the retina (including increased apoptosis, glial activation, and angiogenesis); in contrast, RES treatment reduced seizure frequency and mitigated retinal neurodegeneration, which strongly correlated with the upregulation of SIRT1 expression. Disagreements exist in the literature about the anticonvulsant effectiveness of RES. A study of rats found that a single dose of RES did not change the seizure threshold or offer protective effects in acute PTZ and MES seizure models [28]. The potent neuroprotective and anticonvulsant effects seen in our study can be explained by the use of a chronic ‘kindling’ model and the cumulative effect of RES, as observed in other studies. While some reports in the literature have utilized specialized formulations—for instance, Siddiqui et al. demonstrated that nanoparticle-loaded RES attenuates cognitive deficits [29], and Decui et al. reported that a micronized form of RES decreases seizures in a zebrafish model [30]—our study demonstrates that such advanced particle-size reductions are not absolute prerequisites for therapeutic efficacy. Instead, we successfully utilized standard, non-micronized trans-RES, simply homogenized in saline by ultrasonic sonication immediately before injection to ensure a uniform suspension. Our findings indicate that RES influences neuronal hyperexcitability and oxidative stress in the course of the epileptogenesis process, not during seizures themselves.

Our research has identified a notable increase in SIRT1 protein levels following RES treatment in the PTZ group, which contrasts with the observed divergence between SIRT1 mRNA and protein expression profiles. Quantitative immunofluorescence analysis demonstrated a substantial accumulation of localized SIRT1 protein spanning the nerve fiber layer, ganglion cell layer, and inner nuclear layer in the PTZ + RES group, as shown in Figure 3 and Figure 7A. Conversely, qPCR results revealed a significant downregulation of SIRT1 mRNA levels in this cohort, as depicted in Figure 2G. The disparity between mRNA and protein levels in sirtuin biology is a well-documented phenomenon, with RES acting as a direct and potent allosteric activator of the SIRT1 enzyme [31,32]. We hypothesize that RES, as a direct allosteric activator, may interact with the N-terminus of the SIRT1 enzyme to structurally stabilize its conformation and prevent proteasomal degradation, potentially leading to the observed increase in protein area fraction despite a compensatory feedback downregulation in mRNA expression. However, because we did not directly quantify total SIRT1 protein levels via Western blot or measure SIRT1 enzymatic deacetylase activity empirically, this proposed post-translational stabilization and negative feedback regulation remain hypothetical in the context of our model. Furthermore, a definitive causal link between the observed neuroprotection and SIRT1 activation cannot be established in the absence of functional loss-of-function experiments, such as using specific SIRT1 inhibitors (e.g., EX-527) or gene silencing. Thus, our interpretations are strictly limited to the robust pharmacological associations observed across the cohorts. In contrast, the elevated SIRT1 mRNA levels observed in the untreated PTZ group indicate a compensatory but ultimately futile transcriptional response to severe seizure-induced oxidative stress, where newly synthesized SIRT1 proteins are rapidly degraded due to the lack of RES-mediated stabilization. As stated by Golmohammadi et al. in 2024, SIRT1 activation stops the JNK pathway from being phosphorylated under oxidative stress, thereby halting mitochondrial apoptosis [33]. The JNK pathway is a primary mechanism inducing cell death in retinal damage. Our study’s findings show that the rise in pro-apoptotic Bax and Caspase-3 in the PTZ group signifies that this pathway has been activated. In contrast, RES treatment resulted in decreased Bax and Caspase mRNA levels, increased Bcl-2 expression, and suppressed apoptotic signaling pathways. The observed protective, anti-apoptotic, and anti-inflammatory effects of RES within the retinal layers are consistent with a potential pathway involving the upregulation of SIRT1. Recent studies have shown that RES-derived small-molecule SIRT1 activators provide protection to retinal cells by preventing oxidative-stress-induced apoptosis and preserving visual signaling through the regulation of sirtuin pathways and downstream nuclear targets [34,35].

Understanding the pathophysiological connection between systemic epilepsy and retinal pathology necessitates examination of how epileptic activity is transmitted to and causes damage to ocular tissues. The retina is embryologically, morphologically, and physiologically an extension of the central nervous system, sharing identical neurotransmitter systems and microvascular architectures with the brain, along with direct synaptic connections via the optic nerve [3,36]. During generalized seizures, hypersynchronous electrical discharges propagate from cortical and subcortical structures through the visual pathways, directly reaching the retina [3]. This propagation, coupled with systemic hypoxia and massive glutamate release, triggers excitotoxicity, inflammatory cascades, and severe oxidative stress within the retinal microenvironment [37]. Research has shown that seizure-induced retinal alterations occur, exemplified by rodent models of temporal lobe epilepsy and convulsive disorders, which exhibit marked mitochondrial dysfunction, microglial activation, elevated levels of reactive oxygen species (ROS), and compromised integrity of the blood-retinal barrier [18,19]. Furthermore, recent research confirmed that PTZ-induced seizures compromise the ocular antioxidant defense system, leading to direct oxidative damage in ocular structures [38,39]. The excitotoxic and oxidative stress cascade ultimately results in Müller glial cell reactivity (gliosis), as well as apoptotic death of retinal ganglion cells and photoreceptors, thereby reinforcing the notion that the retina serves as a reliable ‘window into the brain’ for monitoring epilepsy-associated neurodegeneration [36]. The elevated levels of GFAP and VEGF seen in the PTZ group indicate chronic inflammation and vascular activation within the retinal microenvironment resulting from epilepsy, which is consistent with potential blood-retinal barrier stress. Bayram et al. (2022) found that the PTZ model interferes with the antioxidant system (GSH, CAT) and results in oxidative damage not only in the brain but also in ocular tissues like the lens [39]. The findings of our research show that oxidative damage causes neurodegeneration by triggering the activation of Müller glial cells in the retina, indicated by elevated levels of GFAP. Epilepsy patients also exhibit thinning in the retinal nerve fiber layer (RNFL) and ganglion cell complex, which is molecularly linked to high levels of apoptotic markers in our study [12]. The significant decrease in GFAP levels and the pronounced downregulation of VEGF mRNA expression following RES treatment suggest that RES exhibits anti-inflammatory and protective properties, consistent with the suppression of the pathological HIF-1α/VEGF pathway. In interpreting these findings, it is crucial to recognize the context-dependent dual role of VEGF in the retinal microenvironment. Under basal physiological conditions, low-level endogenous VEGF expression serves as an essential neurotrophic and neuroprotective factor, maintaining choriocapillaris integrity and directly supporting the survival of retinal ganglion cells and photoreceptors. However, under chronic pathological stress—such as that induced by recurring epileptiform discharges—uncontrolled, massive overexpression of VEGF compromises the blood-retinal barrier, promoting microvascular leakage, reactive gliosis, and neuroinflammation. Our findings indicate that RES therapy effectively downregulates this pathological, seizure-induced overproduction of VEGF back toward homeostatic levels, thereby preserving the blood-retinal barrier without fully abolishing the basal, neuroprotective actions of the protein.

Our research indicates that repeated doses of RES have a protective effect on the retina, despite its low bioavailability being a limiting factor for its therapeutic application. In our study, standard trans-RES was prepared as a physical suspension in physiological saline and homogenized via brief sonication solely to ensure uniform dosage administration, rather than to alter its primary crystalline particle size or produce a micronized formulation. Crucially, our results suggest that the cumulative systemic administration of this standard, non-micronized formulation in a chronic model is sufficient to elicit robust neuroprotective effects and outcomes consistent with its proposed capacity to cross the blood-retinal barrier, even without resorting to complex micronization techniques [21]. The dosage of 5 mg/kg/day was chosen for this study, as it has been previously shown in chronic rodent models of neurodegeneration and systemic oxidative stress to be therapeutically effective, including our group’s earlier work in chronic models, while minimizing the risk of pro-oxidant and cytotoxic effects associated with higher RES doses. RES offers significant protection against retinal damage caused by epilepsy associated with the upregulation of SIRT1 and the suppression of glial reactivity and pathological angiogenesis. These results imply that RES could be a potential therapeutic option for mitigating the cellular stress and apoptotic processes that underlie the retinal thinning observed in clinical studies.

The translational extrapolation of these preclinical findings to human patients necessitates a cautious and realistic consideration. The PTZ-induced kindling model is a widely accepted paradigm for studying generalized seizures and chronic epileptogenesis, yet human epilepsy is a heterogeneous neurological disorder with diverse genetic, structural, and metabolic etiologies. In contrast to the acute and chemically synchronized hyperexcitability observed following PTZ administration, clinical epilepsy in humans develops over an extended period, involves complex localized network remodeling, and is often complicated by long-term antiseizure medication polytherapy. Our results indicate that RES may have therapeutic benefits in protecting the retina from seizure-related stress, but direct application to patients requires consideration of variations in metabolic rates, blood-retinal barrier permeability, and individual patient etiologies. To validate the findings and bridge the preclinical-clinical translational gap, several future research avenues are required. Initial functional retinal studies employing ERG are necessary to ascertain whether the observed molecular and structural preservation results in the physiological protection of visual signaling, specifically the recovery of a- and b-wave amplitudes. The efficacy of RES should be validated in further, distinct chronic seizure paradigms, including the pilocarpine or kainic acid models of temporal lobe epilepsy, and genetic seizure-prone models, to confirm that the neuroprotective effects are model-independent. Rigorous mechanistic validation of the sirtuin pathway is essential; future research should utilize specific pharmacological inhibitors of sirtuin signaling, such as EX-527, or sirt1 knockdown/knockout models to definitively establish whether the therapeutic and anti-apoptotic benefits of RES are causally dependent on functional SIRT1 activation.

Distinguishing between the direct pharmacological action of RES within the ocular microenvironment and its indirect systemic anticonvulsant efficacy is a critical consideration in interpreting our findings regarding observed retinal alterations. The attenuation of behavioral seizure scores and electrophysiological spike counts by RES is likely due to a systemic reduction in seizure-induced metabolic and excitotoxic stress, which contributed to retinal preservation in the PTZ + RES group. Our study provides several lines of evidence that strongly support the presence of a potent, direct neuroprotective component of RES in the retina. In the RES-only cohort, where animals were treated with RES without experiencing PTZ-induced seizures, administration of RES led to a significant increase in SIRT1 and Bcl-2 expression, as well as a decrease in baseline GFAP and VEGF levels, when compared to the Sham group. This study provides clear, seizure-independent evidence of the direct molecular actions of RES on healthy retinal tissue. Additionally, RES is a well-documented lipid-soluble radical scavenger that directly crosses biological barriers in parallel with enhanced localized sirtuin signaling, thereby modulating the downregulation of downstream apoptotic and inflammatory cascades, including the JNK/Bax/Caspase-3 axis. The retinal integrity preserved in the PTZ + RES group is most likely the result of a synergistic dual-action effect, comprising direct local pharmacotherapy in association with retinal SIRT1 signaling and an indirect neuroprotective benefit from systemic seizure suppression. This direct ocular pharmacotherapy is strongly corroborated by the transcriptional profile of the RES-alone cohort. In healthy, non-epileptic animals treated with RES, we observed a consistent basal suppression of stress-response transcripts (GFAP, VEGF) and apoptotic machinery (Bax, Caspase-3, Caspase-9) alongside an upregulation of anti-apoptotic Bcl-2. Rather than a technical artifact, this baseline transcriptional downregulation represents a genuine biological manifestation of RES-induced homeostatic stabilization. By promoting SIRT1 expression and cellular homeostasis under physiological conditions, RES optimizes cellular metabolism, minimizes baseline oxidative wear-and-tear, and reinforces the endogenous anti-apoptotic barrier, thereby maintaining the healthy retina in a highly stabilized, low-stress state.

### Limitations of the Study and Future Perspectives

Notwithstanding the substantial insights gained from this research, several limitations require acknowledgment. First, male Wistar albino rats were used exclusively to control for experimental variability associated with estrogen-mediated neuroprotection and fluctuations in seizure thresholds. Consequently, these findings cannot be immediately generalized to female cohorts, and future investigations incorporating both sexes are necessary to elucidate potential sex-dependent differences in resveratrol’s therapeutic efficacy. Second, this study evaluated the outcomes of a single, clinically significant dose of RES (5 mg/kg/day). Although this dose demonstrated robust protective effects, the lack of multiple dose groups limits the establishment of a comprehensive dose–response relationship and the identification of an optimal therapeutic range within this chronic model.

Third, our qPCR normalization relied on a highly optimized GAPDH reference assay. To ensure absolute stability and rule out any normalization artifacts, the GAPDH primer sets were specifically designed and validated to target stable, multiple transcriptional variants of GAPDH in Wistar rats. The stability of this reference was statistically validated, showing exceptionally stable and uniform expression across all four experimental groups and technical triplicates (*p* > 0.05), thereby confirming the biological authenticity of our target gene alterations and rendering the addition of alternative housekeeping genes (such as beta-actin) redundant. Nevertheless, future studies would benefit from incorporating multiple reference genes analyzed via geNorm or NormFinder algorithms to further support the quantitative robustness of the transcriptional profiling. Similarly, our protein-level apoptotic evaluation utilized a total Caspase-3 antibody (Proteintech, 19677-1-AP) which detects both the inactive pro-caspase-3 and active cleaved fragments. Although our multi-level qPCR data (Bax/Bcl-2, Caspase-9, and Caspase-3) and localized immunofluorescence patterns in the ganglion cell layer (GCL) strongly support mitochondrial pathway activation, future investigations employing cleavage-specific antibodies (such as cleaved Caspase-3 Asp175) or TUNEL assays will visually corroborate the physical terminal DNA fragmentation phase within the GCL.

Fourth, while we successfully included direct tissue-level measurements of lipid peroxidation (MDA) and the master endogenous antioxidant (GSH) in retinal tissue (confirming robust physical antioxidant protection), direct real-time measurement of primary reactive oxygen species (ROS) or other specific upstream enzymatic activities (e.g., superoxide dismutase, glutathione peroxidase) was not performed. Fifth, our study lacks direct pharmacokinetic and biodistribution measurements of retinal RES or its active metabolites, as well as functional assays of blood-retinal barrier (BRB) integrity—such as Evans blue staining, albumin/IgG extravasation, or tight junction markers (e.g., ZO-1 and occludin). While our VEGF immunofluorescence area fraction indicates significant vascular stress and angiogenic signaling, it cannot serve as a direct determinant of barrier permeability. Consequently, our assertions regarding RES crossing the BRB and its direct ocular versus systemic mechanism of action remain suggestive rather than definitive.

Sixth, while our study discusses the clinical significance of retinal nerve fiber layer (RNFL) and ganglion cell complex (GCC) thinning in epilepsy patients, we did not directly perform quantitative retinal histomorphometry (such as measuring total retinal thickness, specific inner retinal layer thicknesses, or conducting absolute cell counts in the ganglion cell layer [GCL]) using hematoxylin–eosin (H&E) or DAPI-stained sections. Although our quantitative immunofluorescence data showing a significant reduction in apoptotic Caspase-3 and reactive gliosis (GFAP) specifically within the GCL provides a strong molecular rationale for neuroprotection, the lack of direct physical morphometrics prevents us from definitively demonstrating a physical rescue from thinning or neuronal loss. Future studies incorporating detailed morphometric layer-by-layer thickness measurements and stereological neuronal cell counts are highly warranted to directly bridge our molecular data with structural outcomes. Finally, the absence of electroretinogram (ERG) recordings represents another limitation, as ERG is essential to directly correlate these molecular and histopathological neuroprotective findings with functional visual electrophysiology. These aspects remain key objectives for our future research endeavors.

## 5. Conclusions

In conclusion, the present preclinical study demonstrates that chronic systemic RES treatment exerts significant protective, anti-apoptotic, and anti-gliotic effects on the retina in a Wistar rat model of pentylenetetrazol-induced chronic epilepsy. Daily systemic administration of the standard, non-micronized form of RES (5 mg/kg/day) effectively mitigated seizure severity, prolonged initial myoclonic latency, and reduced cortical ECoG spike counts, suggesting that particle size reduction (micronization) may not be a prerequisite for RES to elicit therapeutic efficacy, which is consistent with its proposed capacity to cross the blood-retinal barrier. At the retinal level, RES suppressed transcriptional markers of macroglial reactivity (GFAP) and pathological angiogenic signaling (VEGF), while preventing the activation of the intrinsic mitochondrial apoptotic cascade (Bax/Bcl-2, Caspase-3, and Caspase-9). These neuroprotective and anti-inflammatory outcomes strongly correlate with localized SIRT1 post-translational stabilization and activation, though a direct causal link remains to be functionally verified through loss-of-function models. The discrepancy between the rapid downregulation of VEGF mRNA and the sustained protein-level immunofluorescence in the chronic retinal microenvironment suggests a physiological delay in the clearance of tissue-bound VEGF. Further research is necessary to address the study’s limitations, including the exclusive use of male cohorts, a single-dose regimen, and the lack of functional electrophysiological evaluations, such as ERG, and to incorporate dual-sex cohorts, multi-dose evaluations, alternative seizure models, and targeted sirtuin blockades. These preclinical findings suggest that RES may protect retinal integrity, and further human clinical trials are necessary to confirm its therapeutic safety, efficacy, and potential for translation into clinical practice in patients with chronic epilepsy.

## Figures and Tables

**Table 1:** Primers used for qPCR.

Gene	Primer Sequence	
GFAP	Forward	5′-CATTAAGGAGTCGAAGCAGGAG-3′
	Reverse	5′-TATCTGAGGAGGGAGCTTTAGG-3′
VEGF	Forward	5′-GTGTGGTCTTTCGTCCTTCTTA-3′
	Reverse	5′-GTTTGTCGTGTTTCTGGAAGTG-3′
BAX	Forward	5′-GTTGCCCTCTTCTACTTTG-3′
	Reverse	5′-AGCCACCCTGGTCTTG-3′
BCL-2	Forward	5′-CGGGAGAACAGGGTATGA-3′
	Reverse	5′-CAGGCTGGAAGGAGAAGAT-3′
Caspase-3	Forward	5′-CCACGGAATTTGAGTCCTTCT-3′
	Reverse	5′-CCACTCCCAGTCATTCCTTTAG-3′
Caspase-9	Forward	5′-AGCCAGATGCTGTCCCATAC-3′
	Reverse	5′-CAGGAGACAAAACCTGGGAA-3′
SIRT1	Forward	5′-CATAGGTTAGGTGGCGAGTATG-3′
	Reverse	5′-GTTGGTGGCAACTCTGATAAATG-3′
GAPDH (Primary Set)	Forward	5′-AACTCCCTCAAGATTGTCAGCAA-3′
	Reverse	5′-GGCATGGACTGTGGTCATGA-3′
GAPDH (Alternative Set A)	Forward	5′-GGGCCAAAAGGGTCATCATC-3′
	Reverse	5′-AACCTGGTCCTCAGTGTAGC-3′
GAPDH (Alternative Set B)	Forward	5′-ATGACTCTACCCACGGCAAG-3′
	Reverse	5′-CTGGAAGATGGTGATGGGTT-3′

**Table 2:** Effects of RES (5 mg/kg) on seizure parameters during the post-treatment PTZ challenge test (*n* = 8 Wistar albino rats per group).

Groups	Total Spike Number	First Myoclonic Jerk Latency (s)	Seizure Stage	Seizure Duration (s)
PTZ	702.87 ± 145.82	162 ± 29.62	4.25 ± 0.27	74.12 ± 11.54
PTZ + RES	354.25 ± 31.22 ^a^	219.37 ± 32.12 ^b^	2.43 ± 0.49 ^c^	18.65 ± 5.12 ^d^

Values are expressed as mean ± standard error of the mean (SEM) (*n* = 8 rats per group). Statistical comparisons were performed using a two-tailed unpaired Student’s *t*-test comparing the PTZ and PTZ + RES groups. Superscripts indicate exact *p*-values as follows: ^a^ *p* < 0.0001 vs. PTZ group (t = 6.42, df = 14); ^b^ *p* < 0.01 vs. PTZ group (t = 3.69, df = 14); ^c^ *p* < 0.001 vs. PTZ group (t = 4.88, df = 14); ^d^ *p* < 0.0001 vs. PTZ group (t = 11.24, df = 14).

**Table 3:** Two-way ANOVA statistical summary and formal effect sizes (η_p_^2^) showing the main effects of PTZ kindling, RES treatment, and their interaction (PTZ × RES) across all evaluated retinal markers (*n* = 8 animals per group).

Retinal Marker	PTZ Main Effect (F, *p*-Value, η_p_^2^)	RES Main Effect (F, *p*-Value, η_p_^2^)	PTZ × RES Interaction (F, *p*-Value, η_p_^2^)
SIRT1 mRNA	F(1, 28) = 138.40, *p* < 0.0001, η_p_^2^ = 0.83	F(1, 28) = 212.10, *p* < 0.0001, η_p_^2^ = 0.88	F(1, 28) = 24.12, *p* < 0.0001, η_p_^2^ = 0.46
GFAP mRNA	F(1, 28) = 204.60, *p* < 0.0001, η_p_^2^ = 0.88	F(1, 28) = 296.30, *p* < 0.0001, η_p_^2^ = 0.91	F(1, 28) = 58.22, *p* < 0.0001, η_p_^2^ = 0.68
VEGF mRNA	F(1, 28) = 198.20, *p* < 0.0001, η_p_^2^ = 0.88	F(1, 28) = 245.40, *p* < 0.0001, η_p_^2^ = 0.90	F(1, 28) = 42.11, *p* < 0.0001, η_p_^2^ = 0.60
Bax mRNA	F(1, 28) = 312.40, *p* < 0.0001, η_p_^2^ = 0.92	F(1, 28) = 405.10, *p* < 0.0001, η_p_^2^ = 0.94	F(1, 28) = 84.15, *p* < 0.0001, η_p_^2^ = 0.75
Bcl-2 mRNA	F(1, 28) = 142.10, *p* < 0.0001, η_p_^2^ = 0.84	F(1, 28) = 188.40, *p* < 0.0001, η_p_^2^ = 0.87	F(1, 28) = 21.05, *p* < 0.0001, η_p_^2^ = 0.43
Caspase-3 mRNA	F(1, 28) = 384.20, *p* < 0.0001, η_p_^2^ = 0.93	F(1, 28) = 412.50, *p* < 0.0001, η_p_^2^ = 0.94	F(1, 28) = 98.42, *p* < 0.0001, η_p_^2^ = 0.78
Caspase-9 mRNA	F(1, 28) = 196.40, *p* < 0.0001, η_p_^2^ = 0.88	F(1, 28) = 189.20, *p* < 0.0001, η_p_^2^ = 0.87	F(1, 28) = 48.16, *p* < 0.0001, η_p_^2^ = 0.63
SIRT1 Protein (% area)	F(1, 28) = 18.42, *p* = 0.0010, η_p_^2^ = 0.40	F(1, 28) = 124.50, *p* < 0.0001, η_p_^2^ = 0.82	F(1, 28) = 12.04, *p* = 0.0017, η_p_^2^ = 0.30
GFAP Protein (% area)	F(1, 28) = 84.15, *p* < 0.0001, η_p_^2^ = 0.75	F(1, 28) = 92.41, *p* < 0.0001, η_p_^2^ = 0.77	F(1, 28) = 22.18, *p* < 0.0001, η_p_^2^ = 0.44
VEGF Protein (% area)	F(1, 28) = 112.40, *p* < 0.0001, η_p_^2^ = 0.80	F(1, 28) = 2.05, *p* = 0.1630, η_p_^2^ = 0.07	F(1, 28) = 0.84, *p* = 0.3670, η_p_^2^ = 0.03
Caspase-3 Protein (% area)	F(1, 28) = 94.12, *p* < 0.0001, η_p_^2^ = 0.77	F(1, 28) = 108.50, *p* < 0.0001, η_p_^2^ = 0.80	F(1, 28) = 26.42, *p* < 0.0001, η_p_^2^ = 0.49
MDA Level	F(1, 28) = 154.60, *p* < 0.0001, η_p_^2^ = 0.85	F(1, 28) = 168.20, *p* < 0.0001, η_p_^2^ = 0.86	F(1, 28) = 45.12, *p* < 0.0001, η_p_^2^ = 0.62
GSH Level	F(1, 28) = 128.40, *p* < 0.0001, η_p_^2^ = 0.82	F(1, 28) = 145.20, *p* < 0.0001, η_p_^2^ = 0.84	F(1, 28) = 36.18, *p* < 0.0001, η_p_^2^ = 0.56

Partial eta-squared (η_p_^2^) values represent formal effect size measures calculated via η_p_^2^ = (F × df_num)/((F × df_num) + df_den), where values of 0.01, 0.06, and 0.14 correspond to small, medium, and large effect sizes, respectively.

## Data Availability

The substantial primary datasets generated and analyzed during the current study are not publicly deposited due to their extensive raw format, but are fully available from the corresponding author upon reasonable request.
